# Gene expression variations and allele-specific expression of two rice and their hybrid in caryopses at single-nucleus resolution

**DOI:** 10.3389/fpls.2023.1171474

**Published:** 2023-05-23

**Authors:** Han Zhou, Xing Wang Deng, Hang He

**Affiliations:** ^1^State Key Laboratory of Protein and Plant Gene Research, School of Advanced Agriculture Sciences and School of Life Sciences, Peking University, Beijing, China; ^2^Shandong Laboratory of Advanced Agricultural Sciences at Weifang, Peking University Institute of Advanced Agricultural Sciences, Weifang, Shandong, China

**Keywords:** snRNA-seq, endosperm, rice, caryopsis, development

## Abstract

Seeds are an indispensable part of the flowering plant life cycle and a critical determinant of agricultural production. Distinct differences in the anatomy and morphology of seeds separate monocots and dicots. Although some progress has been made with respect to understanding seed development in *Arabidopsis*, the transcriptomic features of monocotyledon seeds at the cellular level are much less understood. Since most important cereal crops, such as rice, maize, and wheat, are monocots, it is essential to study transcriptional differentiation and heterogeneity during seed development at a finer scale. Here, we present single-nucleus RNA sequencing (snRNA-seq) results of over three thousand nuclei from caryopses of the rice cultivars Nipponbare and 9311 and their intersubspecies F_1_ hybrid. A transcriptomics atlas that covers most of the cell types present during the early developmental stage of rice caryopses was successfully constructed. Additionally, novel specific marker genes were identified for each nuclear cluster in the rice caryopsis. Moreover, with a focus on rice endosperm, the differentiation trajectory of endosperm subclusters was reconstructed to reveal the developmental process. Allele-specific expression (ASE) profiling in endosperm revealed 345 genes with ASE (ASEGs). Further pairwise comparisons of the differentially expressed genes (DEGs) in each endosperm cluster among the three rice samples demonstrated transcriptional divergence. Our research reveals differentiation in rice caryopsis from the single-nucleus perspective and provides valuable resources to facilitate clarification of the molecular mechanism underlying caryopsis development in rice and other monocots.

## 1 Introduction

Seeds of cereal crops greatly contribute to human food, animal feed, biofuels and raw material for manufactured goods. Cereal caryopses mainly consist of three components with distinct genetic origins: filial diploid tissues of the embryo, filial triploid tissues of the endosperm and maternal diploid tissues such as the pericarp, testa, integuments, and nucellus (Lafon-Placette and Köhler, 2014; Liu et al., 2022). In rice, the embryo initiates egg cell fertilization with a sperm nucleus to form a zygote. From the first zygotic division, it takes 10 stages for the single cell (fertilized egg) to develop into the mature embryo, which is marked by landmark events including embryonic shoot apical meristem (SAM) formation, leaf primordia emergence, leaf primordia formation and organ enlargement (Xue et al., 2012). The development of rice endosperms starts from coenocytic nuclear divisions of the triploid central nucleus in the embryo sac after fertilization and continues with cellularization to form cell walls between individual nuclei. The cellular endosperm further differentiates into various tissue types, including aleurone and starchy endosperm, and rapidly accumulates storage products (Liu et al., 2022). The maternal tissues are fused tightly together, encasing the embryo and endosperm. In these maternal tissues, cell division, cell expansion, tissue differentiation and degeneration play important roles in sugar loading and grain filling (Wu et al., 2016; Ingram, 2017).

To more accurately dissect the spatial-temporal partitions of rice caryopses, approaches such as laser capture microdissection (Ram et al., 2021), mechanical squeezing (Luo et al., 2011), glass micropipette aspiration (Kuang et al., 2015), and cell type-specific expression profiling (Birnbaum et al., 2005) have been applied, facilitating the analysis of tissue-specific expression profiles. As a result, a series of genes with tissue-specific expression, transcription factors, and imprinted genes have been systematically identified. Despite such extensive studies, a comprehensive analysis of the identity of every cell in the caryopsis is lacking, limiting our ability to prospectively characterize functions and delineate the differentiation trajectory of cells in rice caryopses. Quantitation of gene expression patterns at the single-cell level is crucial for understanding the development and differentiation process.

Recent advances in single-cell RNA sequencing (scRNA-seq) have revolutionized transcriptomics studies by allowing the detection of transcriptional differentiation at the single-cell level in thousands of cells simultaneously (Grün and van Oudenaarden, 2015). The molecular heterogeneity and developmental progression of tissues are also accurately reflected by scRNA-seq. To reveal dynamic gene expression regulation in plants, scRNA-seq has been successfully applied in protoplasts from diverse cell types, such as roots, shoot apices, leaves, and gametophytes (Shaw et al., 2021). However, this approach is problematic since gene expression noise is created during the protoplasting procedure, and protoplast isolation is difficult or even impossible in certain tissues. To address these difficulties, single-nucleus RNA sequencing (snRNA-seq) has served as an alternative methodology (Habib et al., 2016). Nucleus isolation-based methods have been proven to be applicable to a wide range of plant materials from *Arabidopsis*, including seedlings, roots, inflorescences, embryos, developing endosperm, and seeds, which greatly extend the application prospects of snRNA-seq (Long et al., 2021; Picard et al., 2021).

To date, snRNA-seq of seeds, endosperm, and embryos has mainly been conducted in the dicot *Arabidopsis*, demonstrating distinct transcriptional regulatory mechanisms across emerging cell types. Spatiotemporally heterogeneous gene imprinting, which can potentially explain heterosis at the seed stage, has also been identified (Picard et al., 2021). The seeds in monocots are very different in anatomy and morphology from those in dicots, such as the number and structure of cotyledons and the size of endosperm (Agarwal et al., 2011). In rice, morphological differentiation of the embryo takes place at 4 days after pollination (DAP), and cellularization of the endosperm completes at 5 DAP. 5 DAP is an ideal stage for underlying cell differentiation in rice caryopses. The endosperm determines the quality and yield of rice production and supplies an important food source for humans. Previous research has focused on the molecular mechanism underlying the regulation of rice endosperm development (Vijayakumar and Gowda, 2012; Nie et al., 2013; Zhou et al., 2013; Kuang et al., 2015; Inukai, 2017). In developing rice endosperm, starch mainly accumulates in the central endosperm cells. Other nutrients, such as protein and lipids, tend to be distributed in the aleurone cells on the peripheral endosperm layers (Zhou et al., 2013). However, the molecular mechanisms underlying cell type differentiation and cell fate transition in rice endosperm remain elusive. Although bulk transcriptomics of seeds from various developing stages have been well documented by microarray or RNA-seq, the transcriptome at the early developmental stage at the single-cell level remains largely unknown. Since the most important cereal crops, such as maize, rice, and wheat, are monocots, it is essential to uncover the molecular basis of monocot seed cell types and their differentiation trajectories during seed development at a finer scale.

Allele-specific expression (ASE) is defined as the characteristic of preferentially expressing a particular allele of two alleles in a hybrid (Gaur et al., 2013). One possible mechanism underlying ASE may be parent-of-origin-specific differential expression, which is caused by physiological effects or the imprinting of parental alleles. Under normal circumstances, hybrids evenly express complete sets of chromosomes per nucleus, combining the genetic information from both parents (Tucci et al., 2019); however, epigenetic gene regulatory process violates this rule, resulting in the differential expression of maternally and paternally inherited alleles (Bartolomei et al., 2020). In other words, because of ASE, any given gene could be either a maternally expressed gene (MEG) or a paternally expressed gene (PEG) depending on the preferentially expressed allele. In flowering plants, endosperm has been identified as the major tissue of ASE, while a few ASE genes have been found in the embryo (Yang et al., 2020; Rodrigues et al., 2021). The primary factor controlling the ASE status of many genes is the DNA methylation asymmetries between parental genomes (Picard et al., 2021). Moreover, considering that rice endosperm is a tissue with relatively high heterogeneity, nuclei carrying amplified MEGs are likely intermingled with those carrying amplified PEGs. This heterogeneity among endosperm cell/nucleus types may also account for the varied ASE status in rice endosperm.

In addition, ASE has been suggested to play a key role in the differing levels of heterosis between hybrids and parental lines (Shao et al., 2019; Botet and Keurentjes, 2020). Heterosis refers to the superior phenotypes of hybrids relative to their inbred parents. These superior phenotypes are mostly observed with respect to agronomic traits, including the growth rate, reproductive capacity, yield, and resistance. Utilization of heterosis has greatly promoted agricultural production for over a century (Lippman and Zamir, 2007). There are three classic hypotheses for the genetic mechanisms of heterosis: the dominance and overdominance hypotheses, which are based on allelic interactions, and the epistasis hypothesis, which is based on nonallelic interactions. Other hypotheses, such as the active gene effect, gene network system, and genomic dosage effects, have also been suggested to contribute to heterosis (Huang et al., 2016); however, the genetic and molecular mechanisms underlying heterosis remain elusive and controversial. Previous studies have shown that phenotypic novelty in hybrids is regulated by differential gene expression (Shao et al., 2019). ASE has been proposed to play a role in heterosis in many plants, including *Arabidopsis* (Todesco et al., 2010; Ng et al., 2014; Picard et al., 2021), rice (He et al., 2010; Chodavarapu et al., 2012; Shao et al., 2019), and maize (Springer and Stupar, 2007; Paschold et al., 2012; Paschold et al., 2014).

In rice, there is more genetic variation in intersubspecies hybrids than in intrasubspecies hybrids. The heterosis of intersubspecies is usually stronger than that of intrasubspecies (Zhang, 2020). Based on this information, we chose two completely sequenced cultivars, Nipponbare (*japonica*) and 9311 (*indica*), as parents (Goff et al., 2002; Yu et al., 2002). The F_1_ hybrid was obtained from a cross between female Nipponbare and male 9311. A previous study showed that the hybrid exhibited more days to heading, increased plant height, narrower plant type, increased number of tillers, slightly shorter main panicle length, decreased number of kernels per main panicle, decreased total kernel weight per main panicle, intermediate 1000 grain weight and decreased total grain yield per plant compared to the inbred parents (Venu et al., 2014). Transcriptomic analysis of hybrids and their parents may provide comprehensive insight into gene expression variations and their correlation with heterosis.

Here, we present snRNA-seq results of over three thousand nuclei from rice caryopses of Nipponbare, 9311, and their F_1_ hybrid. A transcriptomics atlas covering most cell types present during the early developmental stage of rice caryopses was constructed, and novel nuclei type-specific marker genes were defined for each cluster. With a focus on rice endosperm, the differentiation trajectory of endosperm subclusters was reconstructed at the single-nuclei level. By profiling ASE in endosperm, 345 genes with ASE (ASEGs) were identified. Further comparisons of differentially expressed genes (DEGs) in each endosperm subcluster among the three samples also demonstrated transcriptional divergence during early endosperm development.

## 2 Materials and methods

### 2.1 Plant materials

The *japonica* cultivar Nipponbare was used as the maternal parent, and the *indica* cultivar 9311 was used as the paternal parent to develop the intersubspecies F_1_ hybrid. All plants were planted in the fields of the Chinese Academy of Agricultural Sciences (Beijing, China) during the normal growth season (May to September) in 2021. The sowing time of these two varieties was calculated by their heading date. To ensure the coordinated flowering process, each rice variety had three sowing dates, separated by 5 days. Plants were emasculated in the morning and pollinated at noon. The young caryopses were harvested 5 days after pollination (DAP); however, the development rate was different between the hybrid and its parents. Nipponbare had already stored a great deal of starch at 5 DAP, whereas the hybrid had just begun to accumulate starch at that time. A portion of the rice caryopses was used to extract DNA for hybrid identification by DNA markers. DNA extraction was performed according to the CTAB (hexadecyl trimethyl ammonium bromide) protocol. The polymorphic InDel marker RID2-1 (an InDel marker in rice) was used. The amplified products were separated by 1.5% agarose gel electrophoresis (Yang et al., 2020).

### 2.2 Nuclear extraction from seeds

The rice endosperm at 5 DAP is a syncytium with partially established cellularization. To overcome the difficulties in constructing protoplasts, we extracted nuclei from rice seeds. The fresh rice seeds were homogenized in Honda buffer (2.5% Ficoll™ 400, 5% dextran T40, 0.4 M sucrose, 25 mM Tris-HCl, pH 7.4, 10 mM MgCI_2_, 10 mM β-mercaptoethanol, and a proteinase inhibitor cocktail) using a mortar and pestle and subsequently passed through a 10-μm (pore-size) filter. Triton™ X-100 was added at a final concentration of 0.5%, and the mixture was incubated on ice for 15 min and centrifuged at 1,500 × g for 5 min at 4°C. The pellet was washed with Honda buffer containing 0.1% Triton™ X-100, gently resuspended in 1 mL Honda buffer, and transferred to a microcentrifuge tube. A 200-μL aliquot of 2.5 M sucrose was placed into a chilled microcentrifuge tube, and 400 μL 60% Percoll^®^ solution in Honda buffer was carefully overlaid. The nucleus-enriched preparation was slowly released onto the side of the tube above the 60% Percoll^®^ layer, after which the tube was centrifuged at 1,000 × g for 20 min at 4°C. The 60% Percoll^®^ layer containing most of the nuclei was carefully removed using a Pasteur pipette, slowly diluted in 1 mL 10× buffer (1% BSA, 0.1% Tween^®^20, 10 mM Tris-HCl, 10 mM NaCl, and 3 mM MgCl_2_), and centrifuged at 800 × g for 10 min to pellet the nuclei. The nuclear pellet was resuspended in 10× buffer to achieve a target concentration of 700–1,200 nuclei/μL.

The caryopses were ground and homogenized in buffer to release nuclei. Then, the impurities were filtered out, and only the nuclei were kept in the precipitate. Since the large amount of starch granules in rice caryopses severely influences nuclear isolation, sucrose density gradient centrifugation was applied to refine the nuclei prior to loading into the 10× Genomics Chromium Controller (Annunziata et al., 2021). The isolated nuclei were mixed with 10× Genomics single nucleus reaction reagents, which included gel bead barcodes. Subsequently, snRNA-seq libraries were generated and sequenced on the high-throughput Illumina platform. Sequencing data were prefiltered at both the cell and gene levels.

### 2.3 snRNA sequencing

The nuclear suspensions were loaded onto a 10X Genomics GemCode Single-cell instrument that generates single-cell Gel Bead-In-Emulsions (GEMs). Libraries were generated and sequenced from the cDNAs with Chromium Next GEM Single Cell 3’ Reagent Kits v3.1. Silane magnetic beads were used to remove leftover biochemical reagents and primers from the post GEM reaction mixture. Full-length, barcoded cDNAs were then amplified by PCR to generate sufficient mass for library construction. R1 (read 1 primer sequence) was added to the molecules during GEM incubation. P5, P7, a sample index, and R2 (read 2 primer sequence) were added during library construction via end repair, A-tailing, adaptor ligation, and PCR. The final libraries contained the P5 and P7 primers used in Illumina bridge amplification. The Single Cell 3’ Protocol was followed to produce Illumina-ready sequencing libraries. A Single Cell 3’ Library comprised standard Illumina paired-end constructs that begin and end with P5 and P7. The Single Cell 3’ 16 bp 10x Barcode and 10 bp UMI were encoded in Read 1, while Read 2 was used to sequence the cDNA fragment. Sample index sequences were incorporated as the i7 index read. Read 1 and Read 2 are standard Illumina^®^ sequencing primer sites used in paired-end sequencing on the Illumina HiSeq2000 platform.

### 2.4 snRNA-seq data processing

snRNA-seq data were aligned to the Nipponbare reference genome (IRGSP-1.0) and counted using Cell Ranger pipelines (version 6.0.1) (Zheng et al., 2017). The raw count matrix data were imported into R using Seurat (version 4.1.1) (Hao et al., 2021) for further analysis. Cells with fewer than 3000 genes and genes detected in fewer than 5 cells were removed. Each individual dataset was scaled and normalized using the ScaleData and NormalizeData functions. The top 2000 highly variable genes were used for principal component analysis (PCA) dimensionality reduction. The first 15 principal components (PCs) were selected according to the PCA elbow plot and used for clustering with a resolution parameter of 0.4. The clusters were visualized and explored by t-SNE and UMAP. Marker genes (cluster-enriched genes) were identified using the function FindAllMarkers. Differential expression analysis was performed based on the Wilcoxon rank-sum test. The cluster-enriched genes were determined using the following parameters: greater than a 1.5-fold difference (logfc.threshold = 0.58) between the two groups of cells and test genes with a minimum fraction of at least 0.1. The cluster-specific marker genes were determined using the following parameters: a log2-fold change greater than 0.25 and a proportion of marker genes expressed in cells among all other clusters (PCT2) of less than 10%. Spearman’s correlation coefficients among the seven identified rice seed clusters and tissue type-specific transcriptomes from two previously published bulk RNA-seq datasets were calculated using the rcorr function in the Hmisc R package.

### 2.5 Integrative analysis of the Nipponbare and hybrid snRNA-seq datasets

Normalized snRNA-seq data from Nipponbare and the hybrid were combined into one dataset. Variable genes were selected using the SCtransform function with default parameters. The FindintegrationAnchors function command was used with default parameters to discover integration anchors across all samples. The IntegrateData function was run on the anchor set with default additional arguments. ScaleData and RunPCA were performed on the integrated assay to compute 12 PCs. UMAP dimensionality reduction was carried out, and a shared nearest neighbor (SNN) graph was constructed using dimensions 1:12 as input features and default PCA reduction. Clustering analysis was performed on the integrated assay at a resolution of 1.

### 2.6 Pseudotime analysis

Pseudotime analysis of rice endosperm differentiation and the determination of cell fate were performed using the Monocle2 (version 2.24.0) R package (Qiu et al., 2017). Dimensionality reduction was performed using the reduceDimension function (reduction_method = ‘DDRTress’), and the cells along the trajectory were ordered using the orderCells function.

### 2.7 Gene ontology analysis

Gene Ontology (GO) is an international standardized gene functional classification system that offers a dynamically updated and controlled vocabulary and a strictly defined concept to comprehensively describe the properties of genes and their products in any organism. GO has three ontologies: molecular function, cellular component, and biological process. The basic unit of GO is the GO term. Each GO term belongs to a type of ontology (Ashburner et al., 2000).

GO enrichment analysis provides all GO terms that are significantly enriched in DEGs compared to the genome background and filters the DEGs that correspond to biological functions. First, all peak-related genes were mapped to GO terms in agriGO v.2.0 with default settings (Tian et al., 2017). Gene numbers were calculated for every term, and significantly enriched GO terms in DEGs compared to the genome background were defined by a hypergeometric test. The formula for calculating the P value is:


$$P=1-\sum_{i=0}^{m-1}\frac{\left(\begin{array}{c} M\\ i \end{array}\right)\left(\begin{array}{c} N-M\\ n-i \end{array}\right)}{\left(\begin{array}{c} N\\ n \end{array}\right)}$$

where N is the number of all genes with GO annotation; n is the number of DEGs in N; M is the number of all genes that are annotated to certain GO terms; and m is the number of DEGs in M. The calculated p values were determined through FDR correction, taking FDR ≤ 0.05 as a threshold. GO terms meeting this condition were defined as significantly enriched GO terms in DEGs. This analysis allowed us to recognize the main biological functions that DEGs perform.

### 2.8 ASE gene identification

Expressed genes with 1,088,928 SNPs between Nipponbare and 9311, previously identified by RiceVarMap2 (Zhao et al., 2021), were used to calculate the maternal, paternal, and total gene expression levels in each rice endosperm nucleus. The allele-specific read count matrixes for rice endosperm were constructed based on these expressed genes. ASE was then assessed using an existing method that accounts for maternal and paternal dosage in endosperm for each gene (single_cell_ASE_analysis.R) (Picard et al., 2021).

### 2.9 Differentially expressed gene identification and classification

DEGs among Nipponbare, 9311, and their F_1_ hybrid were identified using the edgeR package (Robinson et al., 2010). A fold change greater than 1.5 was used as the filtering criterion. DEGs were classified into five major expression patterns based on their expression levels. Moreover, each gene was classified based on its expression level in the F_1_ hybrid versus that in both parental lines. The five patterns were OHP (over-higher parents, F_1_>LP≥3HP), BLP (below-lower parents, F_1_<LP/3≤HP), OMP (over-mid parents, HP<F_1_<3HP), BMP (below-mid parents, F_1_>LP/3 & F_1_<LP), and MPV (similar to mid parents, F_1_>LP & F_1_<3HP). Additive, dominant, and overdominant expressed genes were identified by comparing their expression levels in the hybrid to those in the parents (Li et al., 2016; Liu et al., 2021).

## 3 Results

### 3.1 snRNA-seq and identification of nucleus types in rice caryopses

**Figure 1:**
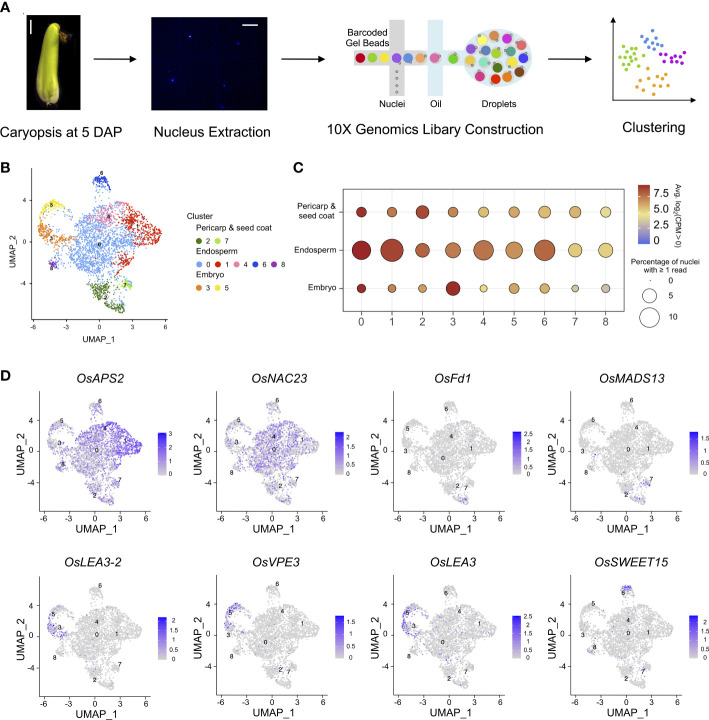
Nuclear heterogeneity in the early developmental stage of rice caryopses. **(A)** Overview of the experimental design. The first panel shows a microscopic image of a Nipponbare caryopsis at 5 DAP (scale bar = 2 mm), and the second panel shows an image of rice nuclei (scale bar = 25 μm). **(B)** Uniform manifold approximation and projection (UMAP) plot of unbiased clustering of the 3,617 nuclei in rice caryopsis. **(C)** Average expression levels of marker genes in the nine identified nuclei clusters in the pericarp and seed coat, endosperm, and embryo. **(D)** UMAP visualization of meta-cluster-specific genes in rice caryopses. The colors represent the normalized gene expression levels, log_10_ (CPM+1), on the UMAP graph.

The rice caryopsis, comprised of the embryo, endosperm, pericarp, and seed coat possessing different ploidy levels, is technically challenging to separate, and profiling of the transcriptome of each tissue type is difficult. To profile transcriptional complexity during the early development of rice caryopses, caryopses at 5 DAP were collected from the *japonica* cultivar Nipponbare, the *indica* cultivar 9311, and their intrasubspecies hybrid obtained from a cross between female Nipponbare and male 9311. A single-nucleus RNA sequencing (snRNA-seq) method was employed to generate a transcriptional atlas of rice caryopses. In total, 3,617 individual nuclei were profiled from these three rice samples, and 20,156, 27,213, and 21,817 expressed genes were detected in Nipponbare, 9311, and their F_1_ hybrid, respectively, using the IRGSP1.0 reference genome (**Figure 1A**; **Table S1**) (Kawahara et al., 2013).

The anatomical structures of the three rice accessions could all be roughly divided into the same three parts (embryo, endosperm and maternal tissues). To this end, snRNA-seq data of Nipponbare, 9311, and their hybrid were normalized to correct for experimental variation. Then, for each nucleus, the genes with the strongest biological signal relative to the technical noise were separately identified based on their expression levels. To reduce the dimensionality of the strongest gene expression features, principal component analysis (PCA) and graph-based clustering analysis [Uniform Manifold Approximation and Projection (UMAP) and *t*-Distributed Stochastic Neighbor Embedding (*t*-SNE)] were performed on the qualifying 3,345 single-nucleus transcriptomes using Seurat (Version 4.1.0) (**Figures 1B**, **S1**). Clusters of nuclei were identified by a shared nearest neighbor modularity optimization-based clustering algorithm. Nine distinct clusters, each containing between 52 and 1,689 nuclei, were identified in the caryopsis.

We noted that three meta-clusters exhibited relatively distinct transcriptomics profiles, and the nuclei clusters in each meta-cluster were grouped on the UMAP and *t*-SNE plots (**Figures 1B**, **S1**). A series of enriched and specific genes for each cluster was identified (**Table S2**). To attribute nuclear identities to these clusters, the expression levels of marker genes whose patterns have been well studied in the endosperm, embryo, pericarp, and seed coat were evaluated for each cluster (**Figure 1C**). The average expression levels of previously reported tissue-specific genes helped us to identify nuclei clusters (Zhou et al., 2013; Wu et al., 2020; Ram et al., 2021) (**Table S3**). The results suggested that clusters 2 and 7 likely represented the pericarp and seed coat, clusters 3 and 5 could be represented the embryo, and the remaining clusters (including clusters 0, 1, 4, 6, and 8) were likely to be the endosperm. The representative cluster marker genes included *OsLEA3-2* (Duan and Cai, 2012), *OsVPE3* (Lu et al., 2016), and *OsLEA3* (Xiao et al., 2007), which are known to be expressed in embryos; *OsFd1* (He et al., 2020) and *OsMADS29* (Yin and Xue, 2012), which are predominantly expressed in maternal tissues (seed coat and pericarp); and *OsAPS2* (Akihiro et al., 2005)*, OsNAC23* (Li et al., 2022), and *OsSWEET15* (Yang et al., 2018), which are important for starch synthesis in the endosperm. These known marker genes were highly and specifically enriched in their corresponding clusters (**Figure 1D**).

Moreover, Gene Ontology (GO) enrichment analysis of the marker genes in each meta-cluster was performed to annotate their putative functions (**Table S4**). For example, genes related to photosynthesis, reproduction, and response to chemical stimuli were enriched in the pericarp like meta-cluster (clusters 2 and 7); genes related to carbohydrate metabolism, pyrophosphatase activity, macromolecule biosynthesis, and protein modification were enriched in the endosperm like meta-cluster (clusters 0, 1, 4, 6, and 8); and genes related to transcription factor activity and RNA biosynthesis were enriched in the embryo like meta-cluster (clusters 3 and 5). GO analysis of each subcluster further demonstrated more detailed functional categories for each nucleus type. In the pericarp like cluster, cluster 2 included specific gene signatures for photosynthesis and signal transmission, while genes highly expressed in cluster 7 are involved in transcription and response to stress. Similarly, for endosperm like clusters, genes involved in lipid metabolism were enriched in clusters 6 and 8, those related to glucan biosynthesis and metabolism and carbohydrate biosynthesis were overrepresented in cluster 1, and those related to nutrient reservoir activity were enriched in cluster 4.

The nucleus type in each cluster was further validated by correlation coefficiency analysis between the cluster-enriched genes and published seed cell-type expression profiles (**Figure S2**) (Wu et al., 2020; Ram et al., 2021). Furthermore, conserved and divergently expressed genes for each homologous cell type were compared among the three rice samples. Venn diagram analysis demonstrated that greater than 70% of hybrid-expressed genes were also expressed in Nipponbare or 9311 in all three meta-clusters. For hybrid-specific genes, the highest percentage was found in the endosperm, which is in accordance with the developmental process of endosperm being the most divergent, while other cell types were more similar between the hybrid and its parents (**Figure S3**).

Taken together, these results show that the nine nuclei clusters yielded two clusters for the embryo like, two clusters for the seed coat and pericarp like, and five clusters for the endosperm like, and reveal a high degree of nuclear transcriptional heterogeneity in rice caryopses.

### 3.2 Construction of an endosperm developmental trajectory

**Figure 2:**
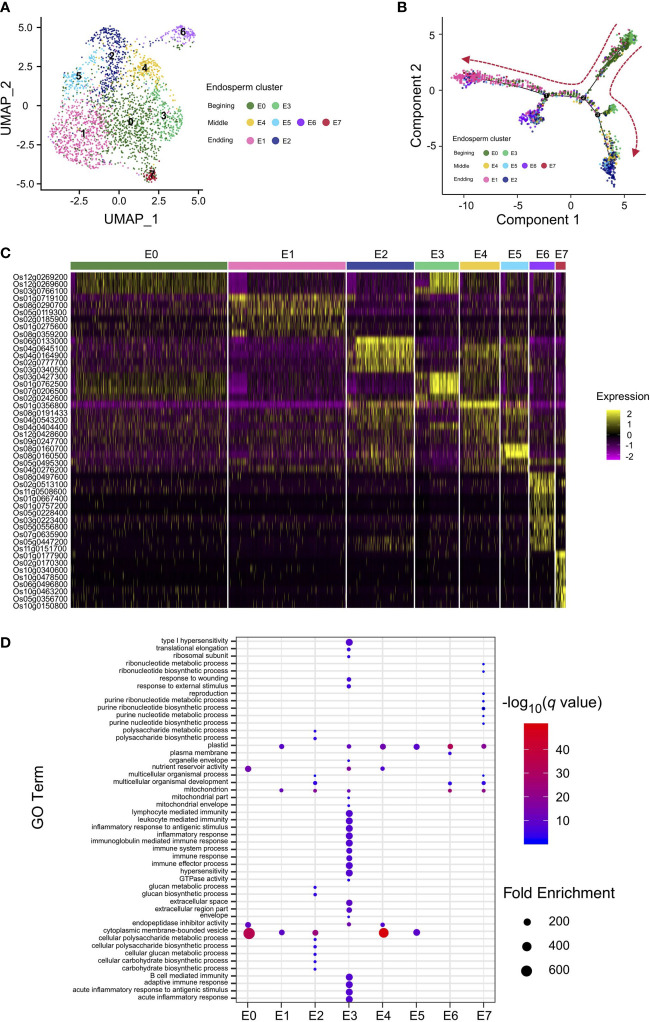
Nucleus type identification and differentiation trajectory reconstruction in rice endosperm. **(A)** UMAP plot of unbiased clustering of the 2,600 rice endosperm nuclei. **(B)** Pseudotime analysis using endosperm nuclei to show the developmental trajectory of rice endosperm. Colored dots indicate nuclei from clusters in **(A)**. **(C)** Heatmap of the scaled expression of markers for each endosperm cluster in all individual nuclei. Nuclei are arranged by cluster. **(D)** Enriched Gene Ontology (GO) terms identified for each cluster according to the cluster-specific genes in each cluster.

To obtain a better understanding of the regulation of rice endosperm development, the corresponding endosperm cells from different stages, including the early stage, differentiated stage, and terminal stage, should be identified. Taking advantage of our snRNA-seq data from rice caryopses, the endosperm nuclear population, consisting of the aforementioned clusters 0, 1, 4, 6, and 8 identified by Seurat, was reclustered. The second subnuclear clustering revealed 8 subclusters from the endosperm, named E0–E7 (**Figure 2A**). The nuclear identities of these endosperm subclusters were determined by examining the enriched expression of marker genes (**Table S5**). The specific enrichment of glucose and starch genes in endosperm subclusters E2, E4, and E5 and translation- and immunity-related genes in endosperm subcluster E3 showed that these two groups of subclusters are hotspot regions for starch and protein biosynthesis, revealing their differentiation in cell fate and storage product preservation during the developmental trajectory. For the remaining endosperm subclusters, genes playing a role in the stress response were enriched in E1, those involved in transducer activity and signal transduction were enriched in E6, and those associated with ribonucleotide metabolism were enriched in E7, indicating potential functions in development and regulation rather than only in storage (**Table S5**).

Interestingly, UMAP analysis revealed that the majority of nuclei from the hybrid endosperm were present in cluster E1, while the proportions of nuclei in clusters E0 and E4 were obviously lower than those from the Nipponbare and 9311 endosperms. The remaining five subclusters were proportionally distributed across all three rice accessions (**Figure S4**). To further clarify the differentiation process in rice endosperm, a developmental trajectory over pseudotime was constructed (**Figures 2B**, **S5**). The expression profiles of the unique marker genes for the eight endosperm subclusters were also compared (**Figure 2C**). At the beginning of the developmental trajectories, nuclei from endosperm subclusters E0 and E3 were specifically distributed, and their expression profiles were enriched in translation, enzyme regulation and endopeptidase inhibitor activity; therefore, they were classified as nuclei where active transcription occurs at the very early stages of endosperm development. Concerning the branch on the right side of the differentiated map, early-stage endosperm subclusters E0 and E3 gradually transitioned into E4 and E2 at the end of the branch, displaying specific expression patterns of glucose and starch synthesis marker genes; therefore, subclusters E2 and E4 were classified as starchy endosperm. Nuclei from subcluster E1, E6 and E7 were mainly grouped at the left of the branch, with marker gene signatures enriched in nitrogen compound metabolic process (**Figure 2D**; **Tables S5**, **S6**), suggesting an active role in the nitrogen compound metabolism. Besides, endosperm transfer cell-containing region-specific gene *AL2* was specifically expressed in E7 (Kuwano et al., 2011). As the aleurone layer adjacent to the vascular bundle would further develop into transfer cells (Thompson et al., 2001), the subcluster E1 were likely to be classified as aleurone layer and E7 to be classified as transfer cells.

### 3.3 ASE of genes from Nipponbare × 9311 in endosperm subclusters

Analysis of expression patterns in the endosperm of hybrids showed that among genes with ASE, the vast majority overexpressed the maternal allele. Maternal traits in plants are predominantly related to seed formation due to the maternal genotype of the endosperm (Botet and Keurentjes, 2020). Therefore, we investigated ASE of genes in endosperm nuclei from the hybrid. We took advantage of the allele-specific nature of our snRNA-seq data to examine ASE across the defined endosperm nuclear subclusters. To achieve reliable allele-specific estimation with low per-cell coverage data, only the 1,088,928 previously reported SNPs between Nipponbare and 9311 from the RiceVarMap2 website were used as a reference for ASE calling. The allele from Nipponbare was deemed the maternal allele, and the allele from 9311 was deemed the paternal allele.

**Figure 3:**
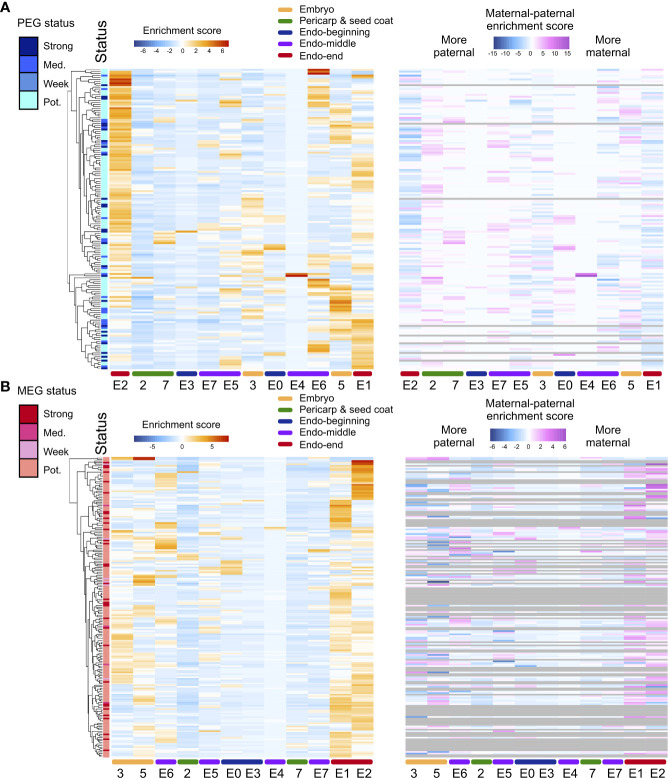
ASE heterogeneity in rice seeds. **(A)** Heatmap of the total expression enrichment score and the difference between the allele-specific maternal and paternal expression enrichment scores for all PEGs in two pericarp and seed coat clusters, one embryo cluster, and six endosperm clusters in the hybrid. **(B)** Heatmap of the total expression ES and ES (maternal) to ES (paternal) for all MEGs. Numbers and characters on the bottom of the figure represent the embryo and pericarp and seed coat, nuclei clusters and endosperm subclusters. Numbers (2, 3, 5 and 7) represent the rice caryopsis clusters except for the endosperm clusters. E0-E7 represent the endosperm subclusters from 0 to 7.

A method for the assessment of ASE that accounts for maternal and paternal dosage in endosperm was employed. Of the 11,426 genes expressed in hybrid endosperm nuclei, ASE was assessed for 2,056 genes. Maternal bias was detected for 189 genes (MEGs), and paternal bias was detected for 156 genes (PEGs). MEGs and PEGS were defined as having strong, intermediate, or weak potential based on the extent of parental bias (**Figure 3**). For MEGs, GO categories were enriched in translation, protein metabolism, protein modification, carbohydrate metabolism, and biosynthesis regulation, while PEGs were enriched in regulatory processes such as the regulation of transcription and RNA metabolism (**Table S8**), which is in accordance with previously reported GO enrichment preferences in rice, maize, and *Arabidopsis* from studies using bulk RNA-seq data (Gehring et al., 2011; Hsieh et al., 2011; Luo et al., 2011; Waters et al., 2011; Chen et al., 2018).

To determine whether the ASEGs were preferentially expressed in specific nucleus types within rice seeds, total and allelic expression patterns were examined across the six newly identified endosperm clusters, two pericarp and seed coat clusters, and one embryo cluster. The heatmap of the total expression of MEGs and PEGs demonstrated that different subclusters of ASEGs were enriched in distinct endosperm nucleus types, with only a few exceptions of common highly expressed genes in several subclusters (**Figure 3**). The highest expression level for both MEGs and PEGs was observed in endosperm subclusters E1 and E2, deemed endosperm nuclei from the late developmental stage. In agreement with this result, endosperm subclusters E0 and E3 showed the lowest expression levels of ASEGs at the beginning of the pseudotime trajectory. Moreover, allele-specific expression values for ASEGs revealed that the enriched expression of MEGs in the specific endosperm clusters was stimulated by increased expression of the maternal allele, whereas expression of the paternal allele remained low and largely unchanged across all endosperm clusters. Expression patterns were the same for PEGs. Essentially, the ASE level is also heterogeneous among endosperm cell/nucleus types.

### 3.4 Gene expression differences in rice endosperm clusters among the hybrid and its parents

**Figure 4:**
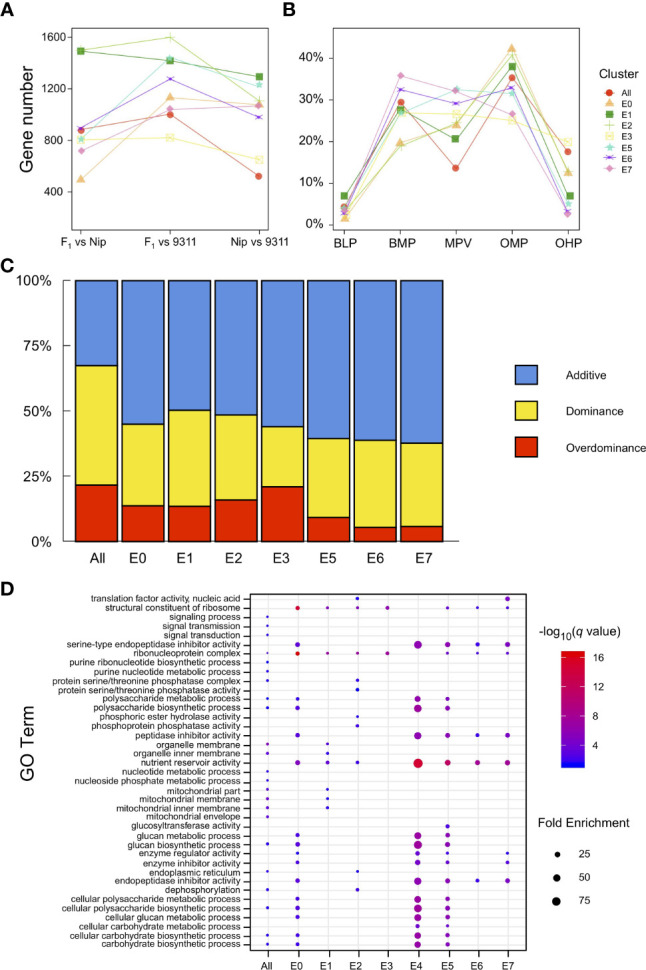
Expression patterns of differentially expressed genes (DEGs). **(A)** Number of DEGs among pairwise comparisons of different endosperm nuclei clusters in Nipponbare, 9311, and their hybrid. **(B)** Ratio of the five major expression patterns (BLP, BMP, MPV, OHP, and OMP) of DEGs in different endosperm nuclei clusters. **(C)** Percentage distribution of additive, dominant, and overdominant genes in each endosperm nucleus type. **(D)** Enriched GO terms identified for each endosperm cluster according to dominant genes.

To explore the differences in gene expression during the early development of rice endosperm at a finer scale, the transcriptome was profiled across the eight endosperm subclusters, and the gene expression levels from the pairwise comparisons among Nipponbare, 9311, and their hybrid were evaluated. A total of 1,468 genes were identified as differentially expressed in at least one pairwise comparison when treating the eight endosperm clusters as a whole, and 1,148–2,503 DEGs were separately detected in single clusters (from E0 to E7, except for E4, owing to only one nucleus being defined as E4 in the hybrid). The late-stage endosperm nuclei clusters E1 and E2 tended to contain more DEGs than the early-stage clusters E0 and E3 (**Figure 4A**). During the process of endosperm development, the determination of nucleus type was accompanied by the identification of a larger set of DEGs. DEGs were classified into five major types based on expression patterns in the hybrid and its parental lines, namely, below-lower parents (BLP), below-mid parents (BMP), similar to mid parents (MPV), over-mid parents (OMP), and over-higher parents (OHP), from low to high relative transcript levels. The results revealed that the expression levels in the hybrid were restricted within the scope of its parents for most of the DEGs. Nevertheless, there were a number of DEGs in the hybrid, ranging from 1.3% to 19.9%, that significantly differed from the lower or higher parent (**Figure 4B**). Of the 1,148–2,503 DEGs with SNPs between Nipponbare and 9311, 3.26–4.25% showed preferential allelic expression of only one maternal or paternal allele.

According to transcriptional changes identified in the hybrid (compared with its parents), DEGs were further classified into additive, dominant, and overdominant. The additive genes were established from early-stage endosperm nuclei E0 and E3 with a high level (55.0–55.9%), reaching a peak in the intermediate stage (60.5–62.2%). Notably, the dominance effects increased with endosperm development, while the overdominance effects varied among early nucleus types and reconciled gradually (**Figure 4C**). GO analysis of the dominant genes in different endosperm clusters demonstrated enrichment in the biosynthesis and metabolism of a variety of substances, such as glucan, nucleotides, carbohydrates, and polysaccharides, and these genes also play a role in nutrient reservoir activity (**Figure 4D**). In contrast, overdominant genes tended to be involved in gene regulation or interactions with other genes (**Table S9**). For example, *RPBF* acts as a transcriptional activator in the regulation of free lysine content and protein accumulation in rice grain (Paponov et al., 2009), and *FLO10* functions by affecting the trans-splicing of mitochondrial *nad1* intron 1, in addition to playing an important role in the maintenance of mitochondrial function and endosperm development (Wu et al., 2019). These results indicate that during rice endosperm development, dominance effects play an important role in substance accumulation, while overdominance effects orchestrate the varied endosperm clusters as a whole.

## 4 Discussion

Rice caryopses display relatively high heterogeneity, with obvious differences in tissue type, ploidy, and source of the genetic material within cells (Itoh et al., 2005). Previous studies have devoted great effort to physically dissecting rice grains using microdissection approaches. Nevertheless, due to significant cell heterogeneity, conventional bulk RNA-seq analysis causes a certain degree of bias by averaging differential gene expression levels among cell populations or overlooking rare cell types (Xue et al., 2012; Shen et al., 2018; Wu et al., 2020; Ram et al., 2021). Owing to the advantages of single-cell techniques, snRNA-seq has been widely applied to study the diversity of transcriptionally distinct cell/nucleus types in many plant tissues, such as developing seeds in *Arabidopsis thaliana* (Picard et al., 2021). In contrast to *Arabidopsis*, knowledge of rice caryopsis development at a fine scale remains limited. We performed the snRNA-seq in rice seeds for the first time. In our study, a transcriptomics atlas of caryopses from the *japonica* cultivar Nipponbare, the *indica* cultivar 9311, and an F_1_ intersubspecies hybrid at 5 DAP was reconstructed at single-nucleus resolution (**Figure 1**). A series of nuclear states were captured during the early stage of rice caryopsis development and assigned to their respective tissue types, including maternal tissue (such as the pericarp and seed coat) and filial tissue (such as the embryo and endosperm), based on well-known marker genes from the literature and bulk RNA-seq results from bioinformatics correlation analysis.

Rice endosperm is one of the most important sources of human food. To date, many molecular studies have identified the important genes required for endosperm development, including the syncytium-cellularization transition, endosperm cell differentiation and storage compound accumulation (Zhang et al., 2021; Liu et al., 2022). In rice interspecies hybrids, the rate of syncytial nuclear division in the endosperm is different from that of their parent lines (Ishikawa et al., 2011). A similar phenomenon was found in our intersubspecies hybrids. Cellularization was not completed in the F_1_ caryopses at 5 DAP. Nuclei extraction rather than protoplast isolation methods were more appropriate to construct scRNA-seq libraries (Wu et al., 2016; Picard et al., 2021). Here, the developmental trajectory of rice endosperm over pseudotime was successfully reconstructed (**Figure 2**). Interestingly, genes required for sugar transportation in grain filling (*OsSWEET11* and *OsSWEET15*) were preferentially expressed in the left branch subclusters (E6 and E7) of the endosperm developmental trajectory (**Figure 2B**; **Table S5**). The highest mRNA levels were detected in regions of the nucellus and the aleurone in a previous study. Considering that the left branch subclusters (E2 and E4) specifically expressed glucose and starch synthesis-related genes, we hypothesized that our snRNA-seq analysis reproduced endosperm cell differentiation in storage product accumulation. In addition, compared with its parents Nipponbare and 9311, a greater number of endosperm nuclei were differentiated to E1 than to E2 in the F_1_ hybrid, and these clusters were concentrated on one of the branches of the developmental trajectory. We propose that the incompatibility caused by interspecies hybridization may influence endosperm development in such a way that it reduces normal nutrient storage. Accordingly, the percentage of nuclei in substance biosynthesis and metabolism-related clusters (E2, E3, and E4) was much lower in the hybrid than in Nipponbare and 9311, and these clusters may critically influence grain filling, cause inadequate energy supply for seed germination, and ultimately lead to a decrease in seed fertility.

Using the intersubspecies hybrid seeds of two rice cultivars (Nipponbare and 9311), we identified 345 genes as ASEGs in the endosperm, displaying deviation in gene expression levels from the predicted 2:1 maternal-to-paternal allele expression ratio (**Figure 3**). Previous research suggests that the functions of imprinted genes, which are important components of ASEGs, are enriched in the synthesis and transport of nutrients. A bulk RNA-seq study in rice endosperm revealed that most of these genes play a role in carbohydrate biosynthesis and metabolism, nitrogen metabolism, and transmembrane transport (Yang et al., 2020). Similar findings were generated in the present study. For MEGs, GO categories were enriched in protein metabolism, protein modification, carbohydrate metabolism, and the regulation of biosynthesis, whereas PEGs were enriched in regulatory processes such as the regulation of transcription and RNA metabolism. These results indicate that ASEGs play an important role in rice endosperm energy metabolism and seed development. Heterogeneity was also found in the expression levels of ASEGs in the endosperm. The highest expression of ASEGs was detected in early developmental stage endosperm, while the lowest expression was detected in the highly differentiated starchy endosperm. These results suggest that the conflict between maternal and paternal alleles is gradually reconciled during seed development. Future cell/nucleus type-specific studies will extend the knowledge of the regulatory mechanism underlying imprinting.

The snRNA-seq data of rice caryopses are likely to include some errors, especially in the discrimination of closely related cell types. Matching the messenger RNA profile of a nucleus with its exact position within a caryopsis is still impossible in this way. Spatial transcriptomics technology can be applied to characterize gene expression in barcoded regions of individual tissue sections (Rao et al., 2021). For plants, spatial transcriptomics has been applied in a limited number of studies. There will be broad application prospects for the *in situ* imaging of mRNAs in future research to directly assay gene expression while preserving positional information. Another major limitation of our work is the lack of evidence from biological experiments. The existing expression data are insufficient to support a causal relationship between gene expression and biological phenomena and are not adequate to resolve the molecular mechanisms underlying heterosis in hybrid rice. Further experiments in which gene functions are validated and phenotypic data of yield-related traits are acquired would improve this research.

In summary, we generated a gene expression map of rice caryopses at single-nucleus resolution. Based on snRNA-seq data, nuclei types from different tissues were divided. We focused on the development, ASE and DEG in endosperm, and these findings would help to utilize inter-subspecies hybridization. The identification of cells in distinct tissue types of the rice caryopsis based on unique transcriptomes now offers researchers the opportunity to investigate, at unprecedented resolution, how the caryopsis develops, how endosperms differentiate, and how ASE affects transcription in hybrid plants.

## Data availability statement

The datasets presented in this study can be found in online repositories. The names of the repository/repositories and accession number(s) can be found in the article/**Supplementary Material**.

## Author contributions

HH carried out project design and material preparation and conceived data interpretation. HZ contributed to the experiments, data analyses, and manuscript writing. All authors contributed to the article and approved the submitted version.
