# Supplementary material for: Gene expression variations and allele-specific expression of two rice and their hybrid in caryopses at single-nucleus resolution
Source: Front Plant Sci. 2023 May 23;14:1171474. doi: 10.3389/fpls.2023.1171474 (PMC10242081; doi:10.3389/fpls.2023.1171474)
Supplement: Supplementary file 1 [file DataSheet_1.docx]

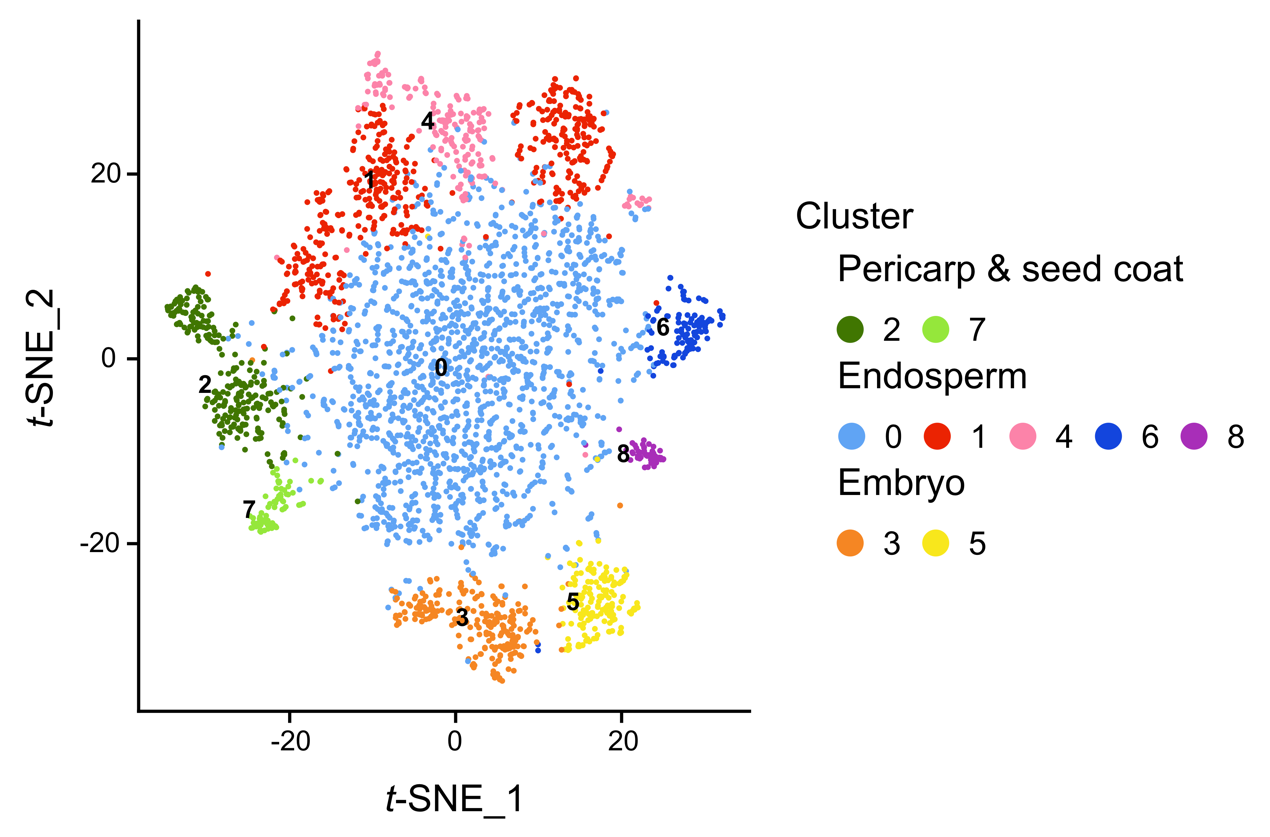
Figure S1. *t*-SNE dimensional reduction of the rice seed nuclei. Nuclei were clustered in nine populations.


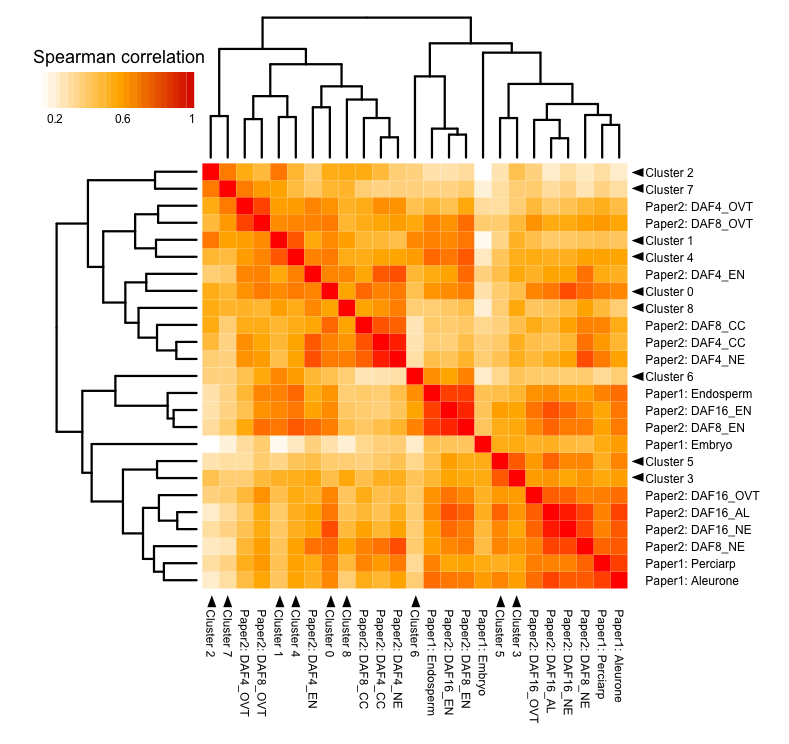
Figure S2. Spearman’s correlation coefficients between our snRNA-seq data with tow previously published bulk RNA-seq papers (DAF: day after fertilization, EN: endosperm, CC: cross cells, NE: nucellar epidermis, AL: aleurone, OVT: ovular vascular).


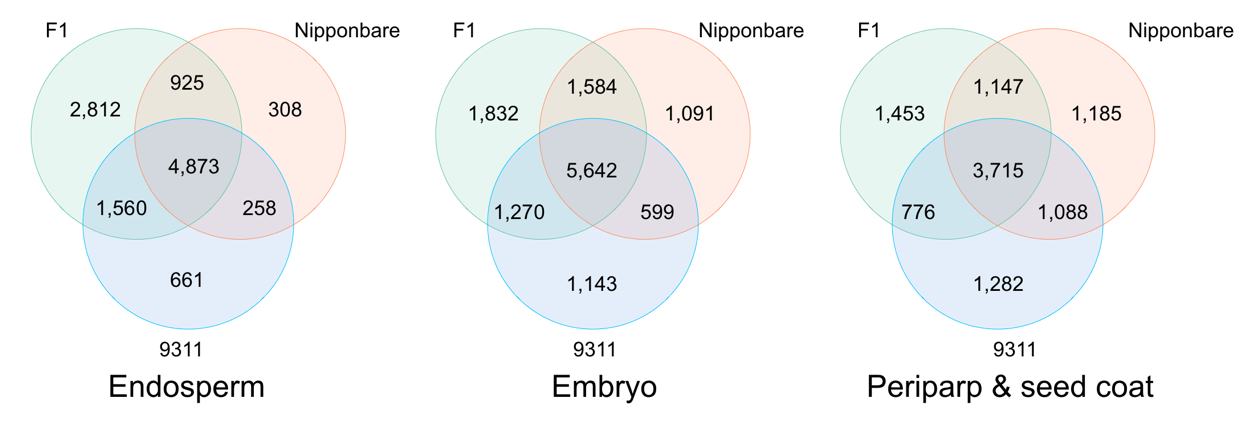


Figure S3. Venn plot for the number of shared and unique expressed genes for the meta-clusters in Nipponbare, 9311 and their F1 hybrid.


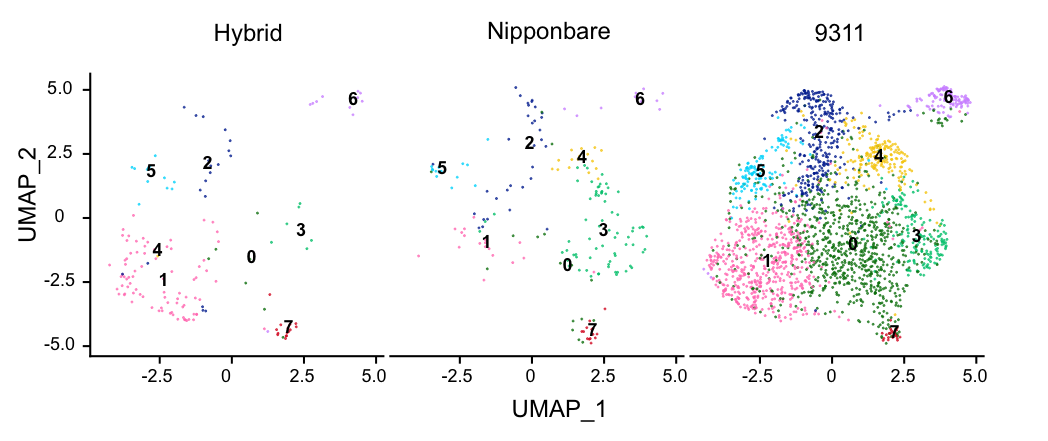


Figure S4. UMAP dimensional reduction of the rice endosperm nuclei in hybrid, Nipponbare and 9311 separately. Nuclei were clustered in eight populations.


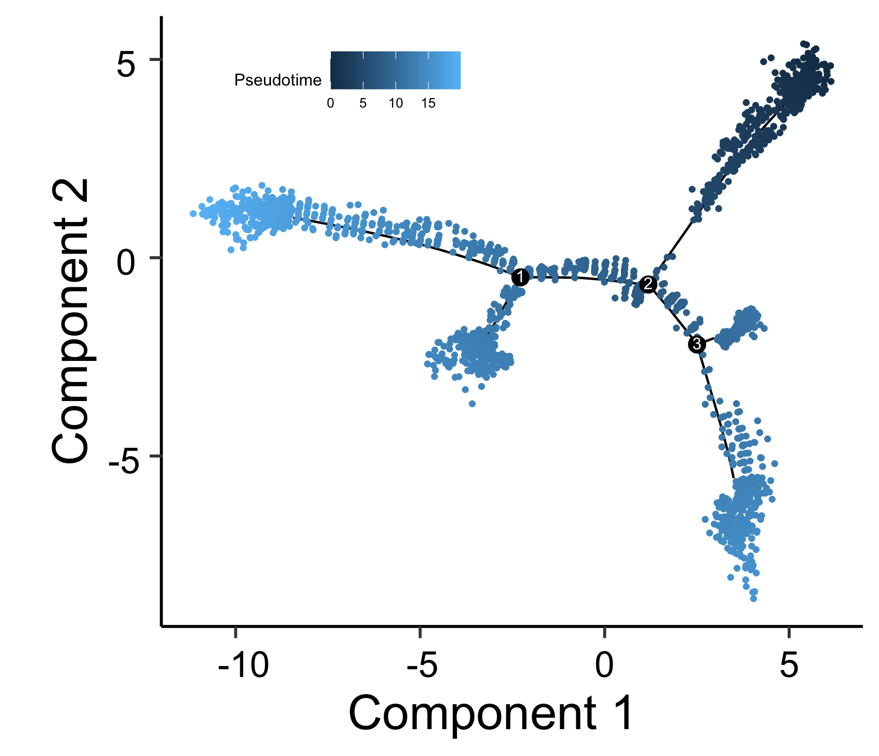


Figure S5. snRNA-seq data of rice endosperm analyzed by Monocle 2, and revealing a key branch point in the trajectory. Each dot indicates a single cell. Color on the dots indicates the pseudotime score.
